# Response to Commentary on “Clinical Predictors of Alzheimer's Disease‐Like Brain Atrophy in Individuals With Memory Complaints”

**DOI:** 10.1002/brb3.71412

**Published:** 2026-04-23

**Authors:** Ahmet Alp Karakasli, Sevilay Karahan, Yavuz Ayhan

**Affiliations:** ^1^ Department of Psychiatry Hitit University Faculty of Medicine Çorum Turkey; ^2^ Department of Biostatistics Hacettepe University Faculty of Medicine Ankara Turkey; ^3^ Department of Psychiatry Hacettepe University Faculty of Medicine Ankara Turkey

1

Dear Dr. Amanda Bischoff‐Grethe and Commentators,

We sincerely thank the Commentators for their meticulous review of our article “Clinical predictors of Alzheimer's disease (AD)‐like brain atrophy in individuals with memory complaints” and for initiating this constructive scientific dialogue. We fully agree that family history of neurocognitive disorders is a critically important factor due to its well‐established strong association with genetic predisposition and early structural brain changes along the AD continuum (R. C. Ratis et al. forthcoming).

The authors have raised a question regarding how the “family history” variable was handled in our multivariable stepwise regression models. In our univariate analyses (Table S5 of the original article [Karakasli et al. 2024]), they noted that family history met our pre‐specified threshold (*p* < 0.25) for inclusion in multivariable models, specifically for the familial AD cortical signature (fAD‐CS, *p* = 0.211) and hippocampal volume (HV, *p* = 0.154) (Karakasli et al. 2024). The authors have rightfully requested an explanation as to why the adjusted results of this variable do not appear in the final models.

We would like to gently draw the authors' attention to Section 3.2 on page 6 of our main text, where the handling of this variable has already been reported: “During the stepwise model selection process, detailed neuropsychological assessment with neuropsychological tests (NPTs), family history, and comorbidity scores were not selected for any dependent variable in at least half of the imputed data sets. Consequently, these variables were not included in the super models” (Karakasli et al. 2024).

To provide the methodological transparency requested by the authors for their ongoing systematic review (PROSPERO: CRD420251089640), we would like to elaborate further (Ratis et al. 2025). Due to missing data elsewhere in our dataset, we applied multiple imputation (fully conditional specification using the mice package) to generate 200 imputed datasets. A strict backward stepwise elimination procedure was then applied to each dataset. For a variable to be retained in the final “super models,” we followed the methodological framework proposed by Wood et al. (2008), which suggests selecting predictors that maintain statistical significance (*p* ≤ 0.05) in at least half of the imputed models (Wood et al. 2008). Although family history exceeded the significance threshold in univariate analysis and had minimal missing data (only two participants), it did not meet this consistency threshold when stronger covariates—such as age, sex, education, and deep white matter ischemic changes—were taken into account.

Therefore, this variable was not manually excluded due to collinearity, nor did it simply “lose significance” in a single static model; rather, its predictive contribution was not consistent enough across 200 iterations to justify inclusion in the final super models. Consequently, there are no meaningful adjusted multivariable coefficients or specific “non‐significant *p*‐values” to report for family history in the final tables.

We appreciate the authors' systematic review efforts and agree that reporting variables that do not survive model selection is important for accurate meta‐analyses and for reducing selective reporting bias. We are happy to share specific raw stepwise data with the authors upon direct request.

Once again, we thank the authors for their shared interest in better understanding preclinical AD and their careful observations.

Sincerely,

The Authors.
